# Supplementary material for: WordCommentsAnalyzer: A windows software tool for qualitative research
Source: F1000Res. 2018 Sep 4;7:536. Originally published 2018 May 3. [Version 2] doi: 10.12688/f1000research.14819.2 (PMC6137411; doi:10.12688/f1000research.14819.2)
Supplement: Supplementary file 3 [file f1000research-7-17614-s0002.tgz › b2a79496-4ada-47c4-9682-a99ce867836d.zip!/Eight Iranian Health Journals/Journal of Health System Research _0.docx]

© Journal of Health System Research . This work is licensed under CC BY-NC 4.0 (https://creativecommons.org/licenses/by-nc/4.0/).

Volume & Issue: دوره 14، شماره 2: 1397 (شماره در حال تکمیل)

Title: بررسی وضعیت پسماندهای تولید شده در مراکز بهداشتی و درمانی استان کهگیلویه و بویراحمد در سال 1394

http://hsr.mui.ac.ir/index.php/jhsr/article/view/3128

مقدمه: افزایش مقدار و تنوع پسماندهای بهداشتی- درمانی، يکي از معضلات مهمی است که سلامت انسان و محیط زیست را تحت تأثیر قرار می‌دهد. به دلیل اثرات عفونی این پسماندها، مدیریت صحیح آن‌ها در مراکز بهداشتی- درمانی ضروری به نظر می‌رسد. هدف از انجام پژوهش حاضر، تعیین کمیت و کیفیت پسماندهای تولیدی در مراکز بهداشتی- درمانی استان کهگیلویه و بویراحمد در سال 1394 بود. روش‌ها: این مطالعه به صورت توصیفی- مقطعی در استان کهگیلویه و بویراحمد انجام گرفت. نمونه‌ها از 432 مرکز بهداشتی- درمانی در استان و به روش سرشماری انتخاب گردید. 3 نمونه در یک سال از واحدهای تابعه دانشگاه علوم پزشکی یاسوج برداشته شد و میانگین آن‌ها به عنوان وزن نهایی اندازه‌گیری شد. داده‌ها با استفاده از نرم‌افزارهای SPSS و Excel مورد تجزیه و تحلیل قرار گرفت. یافته‌ها: کل پسماند تولیدی در واحدهای تابعه دانشگاه علوم پزشکی استان کهگیلویه و بویراحمد، 26/1186 کیلوگرم در روز بود. پسماند‌های معمولی، تیز و برنده، عفونی، شیمیایی، رادیولوژیک و پاتولوژیک به ترتیب 86/80، 10/5، 09/16، 71/0، 36/0 و 44/0 درصد را به خود اختصاص دادند. مراکز بهداشتی- درمانی در شهرستان‌های بویراحمد و چرام به ترتیب بیشترین و کمترین مقدار پسماند عفونی و تیز و برنده را داشتند. نتیجه‌گیری: با توجه به حجم به نسبت بالای پسماند عفونی در مراکز استان (بیش از 16 درصد)، تجهیز مراکز به دستگاه‌های بی‌خطرسازی پسماندهای عفونی و نوک تیز، ضروری به نظر می‌رسد.

Title: بررسی تأثیر ظرفیت کار فیزیکی و جنسیت بر قدرت دست جوانان

http://hsr.mui.ac.ir/index.php/jhsr/article/view/3312

مقدمه: شخص زمانی می‌تواند فعالیت‌های سنگین را راحت‌تر انجام دهد که توان هوازی بیشتری داشته باشد. اندازه‌گیری چنگش دست، راه ارزان و ساده‌ای برای تعیین قدرت ماهیچه است. هدف از انجام مطالعه حاضر، بررسی تأثیر ظرفیت کار فیزیکی (Physical work capacity یا PWC) بر قدرت دست در میان جوانان بود. روش‌ها: این پژوهش به صورت مقطعی بر روی 120 نفر از جوانان 19 تا 29 ساله، با شیوه نمونه‌گیری تصادفی ساده انجام شد. جهت تعیین PWC، قدرت و پایداری چنگش و نیشگون به ترتیب از آزمون پله به روش McArdle، دینامومتر و پینچ‌گیج استفاده گردید. داده‌ها با استفاده از آزمون‌های Independent t و Paired t در نرم‌افزار SPSS مورد تجزیه و تحلیل قرار گرفت. 050/0 > P به عنوان سطح معنی‌داری داده‌ها در نظر گرفته شد. یافته‌ها: قدرت و پایداری چنگش و نیشگون و PWC در مردان بیشتر از زنان بود (050/0 > P). ضمن این که PWC در مردان و زنان به ترتیب فقط بر روی قدرت نیشگون و پایداری چنگش تأثیرگذار بود (050/0 > P). نتیجه‌گیری: به منظور جلوگیری و یا کاهش آسیب‌های جبران‌ناپذیر ناشی از عدم تناسب کار با کارگر، برای آن دسته از مشاغلی که به قدرت نیشگون و پایداری چنگش بالایی نیاز دارند، بهتر است از افرادی با PWC بالا استفاده شود. ضمن این که در به کارگیری افراد در مشاغل مختلف، جنسیت افراد لحاظ گردد.
